# Early Clinical Experience of Finerenone in People with Chronic Kidney Disease and Type 2 Diabetes in Japan—A Multi-Cohort Study from the FOUNTAIN (FinerenOne mUltidatabase NeTwork for Evidence generAtIoN) Platform

**DOI:** 10.3390/jcm13175107

**Published:** 2024-08-28

**Authors:** Atsuhisa Sato, Daloha Rodriguez-Molina, Kanae Yoshikawa-Ryan, Satoshi Yamashita, Suguru Okami, Fangfang Liu, Alfredo Farjat, Nikolaus G. Oberprieler, Csaba P. Kovesdy, Keizo Kanasaki, David Vizcaya

**Affiliations:** 1 Department of Nephrology and Hypertension, International University of Health and Welfare Shioya Hospital, Yaita 329-2145, Japan; 2 Integrated Evidence Generation & Business Innovation, Bayer AG, 13342 Berlin, Germanyfangfang.liu@bayer.com (F.L.);; 3 Medical Affairs & Pharmacovigilance, Bayer Yakuhin Ltd., Breeze Tower, 2-4-9 Umeda, Kita-ku, Osaka 530-0001, Japansatoshi.yamashita@bayer.com (S.Y.); 4 Division of Nephrology, Department of Medicine, University of Tennessee Health Science Center, Memphis, TN 38163, USA; 5 Department of Internal Medicine 1, Faculty of Medicine, Shimane University, 89-1 Enya-cho, Izumo 693-8501, Japan; 6 Center for Integrated Kidney Research and Advance, Faculty of Medicine, Shimane University, 89-1 Enya-cho, Izumo 693-8501, Japan

**Keywords:** chronic kidney disease, diabetes, finerenone, real world, Observational Medical Outcomes Partnership (OMOP), FOUNTAIN

## Abstract

**Background:** In the phase 3 clinical trials FIGARO-DKD and FIDELIO-DKD, finerenone reduced the risk of cardiovascular and kidney events among people with chronic kidney disease (CKD) and type 2 diabetes (T2D). Evidence regarding finerenone use in real-world settings is limited. **Methods:** A retrospective cohort study (NCT06278207) using two Japanese nationwide hospital-based databases provided by Medical Data Vision (MDV) and Real World Data Co., Ltd. (RWD Co., Kyoto Japan), converted to the OMOP common data model, was conducted. Persons with CKD and T2D initiating finerenone from 1 July 2021, to 30 August 2023, were included. Baseline characteristics were described. The occurrence of hyperkalemia after finerenone initiation was assessed. **Results:** 1029 new users of finerenone were included (967 from MDV and 62 from RWD Co.). Mean age was 69.5 and 72.4 years with 27.3% and 27.4% being female in the MDV and RWD Co. databases, respectively. Hypertension (92 and 95%), hyperlipidemia (59 and 71%), and congestive heart failure (60 and 66%) were commonly observed comorbidities. At baseline, 80% of persons were prescribed angiotensin-converting-enzyme inhibitors or angiotensin-receptor blockers. Sodium–glucose cotransporter 2 inhibitors and glucagon-like peptide 1 receptor agonists were prescribed in 72% and 30% of the study population, respectively. The incidence proportions of hyperkalemia were 2.16 and 2.70 per 100 persons in the MDV and RWD Co. databases, respectively. There were no hospitalizations associated with hyperkalemia observed in either of the two datasets. **Conclusions:** For the first time, we report the largest current evidence on the clinical use of finerenone in real-world settings early after the drug authorization in Japan. This early evidence from clinical practice suggests that finerenone is used across comorbidities and comedications.

## 1. Introduction

Chronic kidney disease (CKD) affects two in five people with type 2 diabetes (T2D) worldwide [1]. Globally, approximately 160 million adults were living with CKD and T2D in 2019 [2]. People with CKD and T2D face a significant disease burden including progression to end-stage kidney disease, cardiovascular events, and an increased risk of premature mortality. People with CKD and T2D have a six-times-higher risk of cardiovascular mortality compared to those without CKD or T2D [1,3]. CKD associated with T2D is the biggest cause of end-stage kidney disease in Japan [4].

Until recently, the renin–angiotensin–aldosterone system blockade, with angiotensin-converting-enzyme inhibitors (ACEis) and angiotensin receptor blockers (ARBs), was the sole treatment option for diabetic kidney disease (DKD) [5]. However, the treatment landscape of CKD and T2D is evolving rapidly with the introduction of new agents such as sodium–glucose cotransporter 2 inhibitors (SGLT-2is), glucagon-like peptide 1 receptor agonists (GLP-1 RAs), and finerenone. SGLT-2is were primarily introduced as glucose-lowering therapy; however, numerous clinical trials have established the cardiovascular and kidney benefits of SGLT-2is and established their role in the management of people with diabetic and proteinuric CKD [6,7,8]. The initial use of GLP-1 RAs was to aid in glucose control for people with diabetes [5]. However, a recent clinical trial has demonstrated their cardiovascular benefit and possible kidney benefit, making this medication a new potential option to treat people with DKD [9]. The American Diabetes Association’s Standard of Care in Diabetes recommends a SGLT-2i and a GLP-1 RA as part of the comprehensive cardiovascular risk reduction and/or glucose-lowering treatment plans in people with T2D who have established atherosclerotic cardiovascular disease or kidney disease [10]. Despite the availability of these treatments, people with CKD and T2D continue to have important residual risks for such events [11]. Residual albuminuria after SGLT2i treatment is an independent risk factor for cardiovascular and kidney events [12,13].

Over the past few decades, steroidal MRAs have been used in combination with renin–angiotensin–aldosterone system inhibitors; however, undesired adverse effects, e.g., hyperkalemia, have restricted their usage, prompting the development of nonsteroidal MRAs with better target specificity and safety profiles [14]. Finerenone is a non-steroidal mineralocorticoid receptor antagonist (MRA) that selectively and potently blocks MR overactivation. Pathophysiological mechanisms associated with finerenone include decreasing inflammation, fibrosis, endothelial dysfunction, tissue remodeling, and proteinuria [15]. Mechanisms of finerenone and SGLT-2is are complementary and could provide additional benefits when used concomitantly. In the phase 2 clinical study ARTS-DN Japan [16], when given in addition to a RAS inhibitor, finerenone reduced albuminuria without adverse effects on serum potassium levels or renal function in Japanese patients with T2DM and diabetic nephropathy (DN). In the phase 3 clinical trials FIGARO-DKD and FIDELIO-DKD, finerenone demonstrated cardiorenal benefits in people with CKD and T2D [17,18]. Finerenone was first granted its drug authorization as being indicated for people with CKD and T2D by the Food and Drug Administration in the United States in July 2021 [19]. In March 2022, finerenone was approved in Japan for the treatment of CKD associated with T2D [20]. The European Society of Cardiology guidelines recommend finerenone, in addition to an ACEi or ARB, as a first line of treatment for people with CKD and T2D [21]. Likewise, the European Society of Hypertension guidelines 2023 recommend finerenone because of its nephroprotective and cardioprotective properties in people with diabetic CKD and moderate-to-severe albuminuria [22]. While the beneficial effects of finerenone have been shown in large clinical trials, evidence regarding the real-world use of finerenone, e.g., characteristics, concomitant medications, safety, and effectiveness in routine clinical practice, is now emerging.

Here, we conducted a multi-database retrospective cohort study to generate evidence on the early clinical use of finerenone after its authorization in Japan, and to understand the characteristics, comorbidities, comedications, and occurrence of relevant clinical outcomes in people with CKD and T2D who initiated finerenone in the real world. This study (NCT06278207) was performed as part of the FOUNTAIN (FinerenOne mUltidatabase NeTwork for evidence generAtIoN) platform [23].

## 2. Methods

### 2.1. Study Design, Data Source, and Patient Selection

This was a retrospective cohort study utilizing two Japanese hospital-based databases provided by Medical Data Vision Co., Ltd. (MDV; Tokyo, Japan) and Real World Data Co., Ltd. (RWD Co.; Kyoto, Japan). The MDV database is a hospital claims database collected from 485 acute hospitals in Japan, covering 44 million people in all age groups across whole-country geography. The MDV database comprises health insurance claims data and clinical information recorded in the Diagnosis Procedure Combination (DPC) reporting form [24]. The RWD Co. database is an integrated database of medical information, e.g., electronical medical records, DPC data, and claims collected from 170 hospitals in Japan. The database includes diagnoses, procedures, prescriptions, and laboratory testing results. Both the MDV and RWD Co. databases include hospital diagnoses recorded in inpatient and outpatient care, medical procedures, administration of in-hospital medication, and prescription and laboratory data [25]. The databases used in this study were standardized to the Observational Medical Outcomes Partnership Common Data Model (OMOP-CDM) [26] to ensure reproducibility of the analyzed databases. This study followed relevant European Network of Centers in Pharmacoepidemiology and Pharmacovigilance (ENCePP) guidelines and the International Conference on Harmonization of Technical Requirements for Registration of Pharmaceuticals for Human Use (ICH) guidelines for data management [27,28]. The study period was between 1 July 2021, and 30 August 2023.

**Figure 1:**
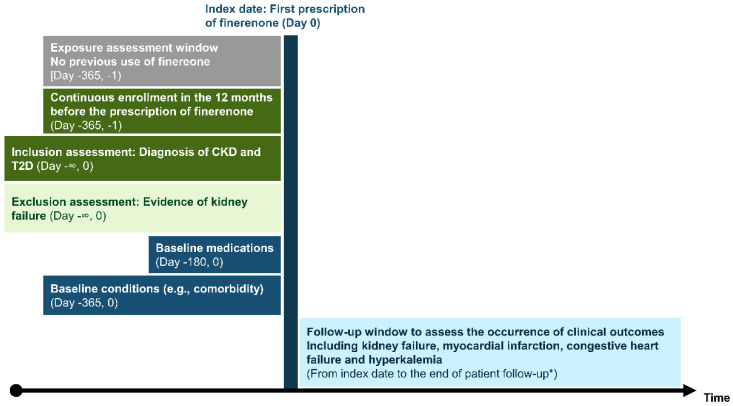
Study design overview. * Persons included in the study were followed up until death, disenrollment from the database, development of kidney failure, or the end of study period, whichever came first. Abbreviations: CKD, chronic kidney disease; and T2D, type 2 diabetes.

Figure 1 shows the overview of the study design. Persons included in the study were identified based on the first record of the prescription of finerenone during the study period. The index date was thus defined as the date of the first prescription of finerenone. We selected people aged ≥ 18 years old, who had prior evidence of T2D, and with CKD stages 2–4. CKD was defined as persons having either a diagnostic code for CKD, two estimated glomerular filtration rates (eGFR) between 15 and 60 mL/min/1.73 m^2^ recorded between 90 and 548 days apart, or two urine creatinine-to-albumin ratio (UACR) measurements of ≥30 mg/g recorded between 90 and 548 days apart at any time before the index date. Type 2 diabetes was defined as persons having at least one diagnostic code for T2D at any time before the index date. Persons were required to have at least 12 months of continuous enrollment in the database and no prior prescriptions of finerenone in the 12 months before the index date. Persons with evidence of kidney failure (eGFR < 15 mL/min/1.73 m^2^, CKD stage 5 or on dialysis, or kidney transplant) at any time before the index date were excluded from the study. Persons were followed until death, disenrollment from the database, development of kidney failure, or the end of study period, whichever came first.

### 2.2. Variables

Demographics, comorbidities, and comedications including treatment for CKD such as an SGLT-2i and a GLP-1 RA were assessed over the 12 months prior to the index date. The information regarding the clinical characteristics related to T2D and CKD including the treatment for T2D and for CKD, diabetes complications, use of insulin, and markers of severity of kidney dysfunction or T2D were collected over 12 months prior to the index date. The CKD stage was categorized based on the KDIGO definition. The Charlson Comorbidity Index and the Diabetes Severity Complication Index were calculated based on a previously reported method [29,30,31]. The list of variables used to assess eligibility, comorbidities, and comedications of the study population can be found in Supplementary Table S1. The information regarding clinical outcomes including kidney failure, acute myocardial infarction (MI), congestive heart failure (CHF), and a cardiovascular composite outcome (MI and CHF) was collected during the follow-up period. Likewise, the occurrence of “confirmed” hyperkalemia was assessed using laboratory data in combination with diagnostic codes of hyperkalemia based on the previous study to assess the incidence of hyperkalemia using a large administrative claims database [32]. The detailed definitions of clinical outcomes are listed in Supplementary Table S2.

### 2.3. Statistical Analyses

Continuous variables were summarized using the mean, standard deviation (SD), median, and first and third quartiles (Q1, Q3). Categorical variables were summarized using counts (n) and percentages (%). Missing data were not imputed. The absolute and relative number of missing values was calculated and reported. The analyses were performed in the overall study population and stratified by baseline use of selected drug classes, i.e., persons treated with an SGLT-2i and/or a GLP-1 RA. Persons could be assigned to multiple subgroups if they took treatments from different drug classes during the baseline period. A subgroup analysis was also conducted in persons with a history of CHF. The stratifications of subgroups were based on the record between day −90 and day 30, which was different from the assessment periods for comedications and comorbidities. The assessment of kidney failure, MI, and CHF required outcome-specific exclusion criteria to ascertain the de novo occurrence of outcomes. For the ascertainment of MI, persons with evidence of a MI at any time before or on the index date were excluded. Likewise, persons with a history of CHF and a history of kidney cancer were excluded from the outcome ascertainment of the CHF and kidney failure, respectively. The main analysis for the incidence of hyperkalemia was based on one serum potassium measurement of >5.5 mmol/L or one diagnosis code of hyperkalemia based on the previous report. A sensitivity analysis was also performed for hyperkalemia using a definition based on the combination of laboratory test results, diagnostic codes, and the use of potassium binders. All statistical analyses were performed using the Observational Health Data Science and Informatics (OHDSI) tools ATLAS v.2.13.0 [33] and R v.4.2.3 [34]. This study followed the STROBE (Strengthening the Reporting of Observational Studies in Epidemiology) statement [35], which is reported in Supplementary Table S3.

### 2.4. Ethics Statement

Because this study utilized only de-identified secondary data, informed consent was waived in accordance with the Ethical Guidelines for Medical and Health Research Involving Human Subjects [36]. The use of de-identified data followed the local regulations including the Personal Information Protection Law. This study protocol was reviewed and approved by the independent ethics committee (MINS: MINS-REC-240209).

## 3. Results

### 3.1. Baseline Characteristics at Finerenone Initiation in the Main Cohort

**Figure 2:**
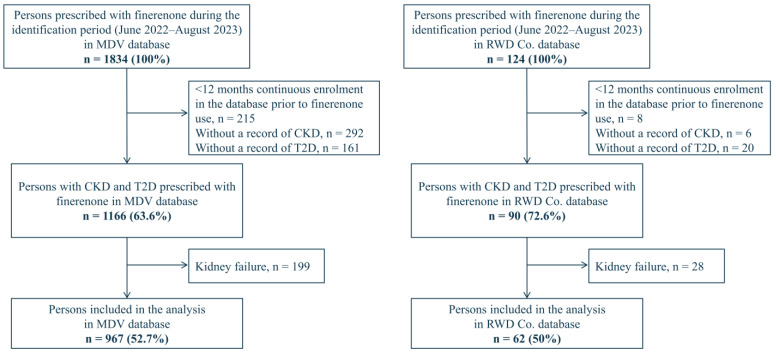
Flow diagram of persons included in the study. Abbreviations: CKD, chronic kidney disease; MDV, Medical Data Vision; RWD Co., Real World Data Co. Ltd; and T2D, type 2 diabetes.

We identified 1834 and 124 individuals initiating finerenone during the study period in the MDV and RWD Co. databases, respectively. After excluding 215 and 8 persons with <12 months of continuous observation before initiation of finerenone, 292 and 6 persons without CKD records, 161 and 20 persons without T2D records, and a total of 1161 (63.6%) and 90 (72.6%) persons met the inclusion criteria, of which, 199 and 28 persons with kidney failure prior to the index date were excluded. Finally, 967 (52.7%) and 62 (50%) persons were included in the analysis (Figure 2).

Table 1 shows the baseline characteristics in the finerenone-initiator cohorts of persons with CKD and T2D. The mean (SD) age was 69.5 (12.4) and 72.4 (10.4) years in the MDV and RWD Co. databases, respectively. A total of 27% were female. Most of the study population had CKD stages 3–4 based on the classification using eGFR records in the RWD Co. database (stage 2: 3.2%, stage 3: 53.2%, and stage 4: 43.6%; n = 62). The mean (SD) eGFR was 43.4 (18.1) mL/min/1.73 m^2^. The UACR categories were as follows: A1 < 30 mg/g, 6.5%; A2 30–300 mg/g, 19.4%; A3 > 300 mg/g, 9.7%; and no assessment in the year before the index date, 62.9%. Among those with at least one record available at baseline, the median (Q1, Q3) UACR closest to the index date was 267.8 (53.3, 300) mg/g. A total of 854 (88.3%) patients were prescribed finerenone 10 mg as a first observed dose (“starting dose”) and 113 (11.7%) patients were prescribed 20 mg. Commonly observed comorbidities included hypertension (92.5% and 95.2%), hyperlipidemia (58.7% and 71%), and CHF (59.7% and 66.1% in the MDV and RWD Co. databases). Among the persons with CHF, 16–22% had a history of CHF hospitalization within the 12 months prior to the index date. A total of 18.3% and 25.8%, and 22.2% and 24.2% persons had a history of acute coronary syndrome and cerebrovascular disease in the MDV and RWD Co. databases, respectively. Retinopathy and neuropathy were observed in 15–20% of the study population. The Charlson Comorbidity Index and the Diabetes Complication Severity Index were slightly higher in persons identified in the RWD Co. database than those in the MDV database.

Baseline comedication use was common, with 80% of individuals using an ACEi or an ARB, one-third of persons using a beta-blocker, and a half of persons using calcium-channel blockers. Before finerenone initiation, 10–20% of individuals were prescribed mineralocorticoid receptor antagonists other than finerenone. Approximately 96% of finerenone users used anti-hyperglycemic medications, including dipeptidyl peptidase 4 inhibitors in 50–65%, metformin in 33–34%, and insulins in 27–30% of persons. At baseline, 72% of persons were prescribed an SGLT-2i in both datasets. Likewise, approximately 30% of individuals were prescribed a GLP-1 RA.

### 3.2. Baseline Characteristics in the Subgroups

Table 2 shows the baseline characteristics in the subgroups of finerenone-initiator cohorts of persons with a concomitant use of an SGLT-2i, a GLP-1 RA, or both an SGLT-2i and a GLP-1 RA. Compared with the overall population of finerenone initiators (n = 967 in the MDV database and n = 62 in the RWD Co. database), persons with a concomitant use of an SGLT-2i (n = 676 in the MDV database and n = 38 in the RWD Co. database) were slightly younger (mean age of 68.1 and 71.8 years in the MDV and RWD Co. databases, respectively). In this subgroup, 30% of persons also concomitantly used a GLP-1 RA. In the subgroup of finerenone initiators with concomitant use of a GLP-1 RA (n = 256 in the MDV database and n = 15 in the RWD Co. database), the mean baseline glycated hemoglobin (HbA1c) levels were higher compared to the overall study population of finerenone initiators. In this subgroup, relatively high proportions of individuals were treated with insulin. Also, in the subgroup of persons with concomitant GLP-1 RA use, approximately 80% of individuals concomitantly used an SGLT-2i. In the subgroup of persons with CHF (n = 508 in the MDV database and n = 33 in the RWD Co. database), the mean age was slightly higher compared with the overall study population of finerenone initiators (Supplementary Table S4). Atrial fibrillation and a history of acute coronary syndrome were more prevalent than in the overall study population. In this subgroup of persons with CHF, relatively high numbers of individuals were treated with SGLT-2is (73–77%), beta-blockers (49–55%), and angiotensin-receptor blocker/neprilysin inhibitors (21–33%). A total of 15–26% of individuals were already treated with other steroidal or non-steroidal MRAs prior to the initiation of finerenone.

### 3.3. Incidence of Clinical Outcomes after Finerenone Initiation

Table 3 shows the incidence of hyperkalemia after finerenone initiation. The median (Q1, Q3) durations of follow-up were 52 (27, 86) and 42 (15, 98) days in the MDV and RWD Co. databases, respectively. The incidence proportions of hyperkalemia were 2.16 and 2.70 per 100 persons in the MDV and RWD Co. databases, respectively. There were no hospitalizations associated with hyperkalemia observed in either of the two datasets. Due to the short follow-up time, very few cases had cardiovascular or kidney failure outcomes. The incidence of these clinical outcomes can be found in Supplementary Table S5. Likewise, the results of incidence of clinical outcomes in the subset of individuals with CHF are reported in Supplementary Table S6. In the sensitivity analysis for hyperkalemia, the incidence proportions of hyperkalemia were 2.16 and 2.63 per 100 persons in the MDV and RWD Co. databases, respectively (Supplementary Table S7).

## 4. Discussion

In this contemporary study, we report on the early clinical use of finerenone in real-world settings, just after drug authorization in Japan. This study provides new population-based insights into the clinical use of finerenone in Japan, adding to the previously reported case series in persons with CKD stage 4 and treated with finerenone [37,38,39], with a broad range of individuals with CKD and T2D included in the study.

The findings from this study suggest finerenone was used across individuals with a broad range of characteristics. Compared to the persons enrolled in the phase 3 clinical trials [17,18,40], persons in this study tended to be older. Similar to the previous study reporting the characteristics of the medication-initiator cohort of finerenone in the United States [41], approximately 40% of prescriptions were given to persons at the age of 70 years. One fifth of the study population were not prescribed an ACEi or ARB within 6 months prior to finerenone initiation; however, some of these individuals may have been treated with a angiotensin-receptor blocker/neprilysin inhibitor. Since the end of the phase 3 clinical trials and with an evolving standard of treatment, we observed a higher proportions of persons concomitantly treated with an SGLT-2i and a GLP-1 RA at finerenone initiation. Notably, an SGLT-2i was prescribed in approximately 70% of the main study population, suggesting that the use of cardio- and reno-protective medication has become a widely recognized option to treat people with DKD.

The KDIGO 2024 clinical practice guideline for the evaluation and management of CKD recommends a comprehensive approach to improve cardiovascular and kidney outcomes in people with CKD and diabetes [42]. This approach includes a foundation of lifestyle modification and self-management for all persons, upon which first-line drug therapies are layered according to the clinical characteristics (metformin, an SGLT-2i, renin–angiotensin–aldosterone system blockade, and a statin), additional drugs with proven kidney and heart protection, as guided by assessments of residual risks, and additional interventions are used as needed to further control risk factors including lipid management, glycemic control, and blood pressure control. For the treatment of people with multiple complications, it is important to pay sufficient attention to signs of worsening kidney function and consider the early initiation of treatment for kidney protection. Despite the established efficacy and safety in reducing the risk of cardiovascular and kidney events and slowing down kidney function decline of the guideline-recommended pharmacological therapies demonstrated in the clinical trials [17,18], these therapies are not fully implemented in real-world settings for a variety of reasons [43].

Organ-protective treatment has been increasingly integrated into clinical practice for people with CKD and T2D. The Japanese Clinical Practice Guideline for Diabetes 2024 by the Japanese Diabetes Society recommends finerenone alongside ACEis or ARBs, SGLT-2is, and a GLP-1 RA for cardio- and renoprotective treatment to reduce the progression of nephropathy for people with DKD [44]. We observed that approximately 70% of persons in this study had comedication of an SGLT-2i in both datasets, suggesting that finerenone is independently introduced to other treatments for heart and kidney protection with an expectation of a synergic effect from the different modes of action. In this study, a low incidence of hyperkalemia was observed within a short follow-up time after finerenone initiation. The incidence of hyperkalemia was also low in the CHF subgroup. The findings were consistent with those of the phase 3 clinical trials [17,18], reporting that 3.1% and 0.5% of persons in the overall finerenone group and 1.2% and 0.1% of persons in the placebo group had serum potassium levels of >5.5 and >6.0 mmol/L, respectively, at one month [45]. Several clinical and observational studies of MRA users in people with renal disease observed an increase in serum potassium levels, with some of these studies observing an increase during the first 3 months of therapy initiation [46,47]. Although these results come from a small sample size with a short follow-up time, these findings may indicate a low rate of hyperkalemia in clinical practice, which is in line with the findings from the phase 3 clinical trials.

This study has several strengths. This study utilized multiple data sources to describe the use of finerenone in a broad range of real-world settings. This is the first, hence the largest, population-based real-world evidence study in Japanese finerenone initiators. The common data structure based on the OMOP-CDM with the same analytical protocol ensures the reproducibility of the analyses conducted across different datasets. Consistent findings in the baseline characteristics and the incidence of clinical outcomes support the reliability of the findings from this study, despite the small-to-moderate sample size of the study population identified in each dataset. Several limitations should also be noted. The relatively small sample size as well as the short follow-up times after finerenone initiation, due to the limited availability of data after the drug authorization of finerenone in Japan, restricted the robust estimation of the incidence of clinical outcomes. This had a greater effect on data from the RWD Co. database, since it was a smaller dataset with fewer hospitals included, and the data cutoff due to the data time lag for the majority of hospitals was several months earlier than for the MDV database after the prescription limitation of 2 weeks was lifted in June 2023. The limited testing of urine in routine clinical care led to an insufficient evaluation of UACR in the RWD Co. dataset. Studies have shown that combining eGFR and UACR levels is more accurate when predicting the risk of CKD progression and cardiovascular disease or mortality in people with T2D. While eGFR screening is widely utilized, the rates of UACR testing in people with T2D remain suboptimal [48]. Despite not being able to assess UACR reduction in Japan, a recent US study observed a ~40% reduction in UACR from baseline at 4 months, which was sustained after 12 months [49]. Although the MDV and RWD Co. databases cover broad ranges of hospitals across geographies in Japan, they did not capture the clinical information collected in primary care settings such as by general practitioners. Despite these limitations, this study provides the first insights into the real-world use of finerenone in Japan, e.g., the characteristics of individuals treated with finerenone and the occurrence of hyperkalemia early after the initiation of the treatment. Further research in larger cohorts of Japanese users of finerenone is warranted. It should also be noted that the study used the Japanese databases; therefore, the findings may not be applicable to the other countries. Further research in diverse populations is needed to validate these findings globally.

## 5. Conclusions

For the first time, we report the largest current evidence on the clinical use of finerenone in real-world settings, early after the drug authorization in Japan. This early evidence from clinical practice suggests that finerenone is used across comorbidities and comedications. The incidence of hyperkalemia after finerenone initiation appears low; however, further studies are required to confirm the safety and effectiveness of finerenone in people with CKD and T2D in routine clinical practice.

## Figures and Tables

**Table 1:** Baseline characteristics in the medication-initiator cohorts of persons with CKD and T2D who initiated finerenone.

	MDV (N = 967)	RWD Co. (N = 62)
Age (years)		
Mean ± SD	69.5 ± 12.4	72.4± 10.4
Median (Q1, Q3)	72 (62, 79)	74 (67, 80)
Gender, female, n (%)	264 (27.3)	17 (27.4)
Index year, n (%)		
2022	101 (10.4)	28 (45.2)
2023	866 (89.6)	34 (54.8)
Hemoglobin A1c, %		
Mean ± SD	7.4 ± 1.5	7.3 ± 1.1
Median (Q1, Q3)	7.0 (6.4, 8)	7.0 (6.5, 7.8)
Missing, n (%)	827 (85.5)	1 (1.6)
eGFR, mg/min/1.73 m^2^		
Mean ± SD	–	43.4 ± 18.1
Category, n (%)		
Stage 2 60–89	–	2 (3.2)
Stage 3 30–59	–	33 (53.2)
Stage 4 15–29	–	27 (43.6)
Finerenone dose initiation, n (%)		
10 mg	854 (88.3)	58 (93.6)
20 mg	113 (11.7)	4 (6.5)
Comorbidity, n (%)		
Hypertension	894 (92.5)	59 (95.2)
Hyperlipidemia	568 (58.7)	44 (71.0)
Congestive heart failure	577 (59.7)	41 (66.1)
Prior hospitalization for heart failure	212 (21.9)	10 (16.1)
Coronary heart disease	372 (38.5)	23 (37.1)
Peripheral vascular disease	137 (14.2)	14 (22.6)
Atrial fibrillation	154 (15.9)	10 (16.1)
Acute coronary syndrome	177 (18.3)	16 (25.8)
Myocardial infarction	87 (9)	8 (12.9)
Cerebrovascular disease	215 (22.2)	15 (24.2)
Neuropathy	198 (20.5)	9 (14.5)
Retinopathy	149 (15.4)	9 (14.5)
Charlson Comorbidity Index		
Mean ± SD	8.2 ± 3.1	10.2 ± 3.5
Median (Q1, Q3)	8 (6, 10)	9 (8–12)
Diabetes Complication Severity Index		
Mean ± SD	5.2 ± 1.8	6.7 ± 2.1
Median (Q1, Q3)	5 (4, 6)	6 (5, 8)
Comedications, n (%)		
ACEi or ARB	776 (80.3)	50 (80.7)
ACEi	350 (36.2)	27 (43.6)
ARB	717 (74.2)	45 (72.6)
ARNI	208 (21.5)	10 (16.1)
Calcium-channel blockers	481 (49.7)	30 (48.4)
Beta-blockers	297 (30.7)	22(35.5)
Loop diuretics	218 (22.5)	17 (27.4)
Thiazide diuretics	45 (4.7)	2 (3.2)
Steroidal MRA	143 (14.8)	5 (8.1)
Non-steroidal MRA other than finerenone	64 (6.6)	1 (1.6)
Statins	571 (59.1)	44 (71.0)
Anticoagulants	153 (15.8)	11 (17.7)
Potassium binders	62 (6.4)	2 (3.2)
SGLT-2i	694 (71.8)	45 (72.6)
GLP-1 RA	268 (27.7)	19 (30.7)
SGLT-2i or GLP-1 RA	760 (78.6)	48 (77.4)
SGLT-2i and GLP-1 RA	202 (20.9)	16 (25.8)
Metformin	322 (33.3)	21 (33.9)
Dipeptidyl peptidase 4 inhibitors	480 (49.6)	40 (64.5)
Sulfonylureas	133 (13.8)	16 (25.8)
Meglitinides	122 (12.6)	13 (21)
Alpha-glucosidase inhibitors	113 (11.7)	4 (6.5)
Thiazolidinediones	30 (3.1)	1 (1.6)
Insulins	295 (30.5)	17 (27.4)

Abbreviations: ACEi, angiotensin-converting-enzyme inhibitor; ARB, angiotensin-receptor blocker; ARNI, angiotensin-receptor blocker/neprilysin inhibitor; CKD, chronic kidney disease; eGFR, estimated glomerular filtration rate; GLP-1 RA, glucagon-like peptide 1 receptor agonist; MRA, mineralocorticoid receptor antagonist; SD, standard deviation; and SGLT-2i, sodium–glucose cotransporter 2 inhibitor.

**Table 2:** Baseline characteristics in the subgroups of medication-initiator cohorts of persons with CKD and T2D who initiated finerenone with the concomitant use of an SGLT-2i, a GLP-1 RA, or both an SGLT-2i and a GLP-1 RA.

	MDV			RWD Co.		
	SGLT-2i (N = 676)	GLP-1 RA (N = 256)	SGLT-2i and GLP-1 RA (N = 187)	SGLT-2i (N = 38)	GLP-1 RA (N = 15)	SGLT-2i and GLP-1 RA (N = 10)
Age (years)						
Mean ± SD	68.1 ± 12.9	65.2 ± 12.5	63.4 ± 12.3	71.8 ± 10.0	71.9 ± 8.5	71.8 ± 8.3
Median (Q, Q3)	71 (59, 77)	67 (57, 75)	65 (55, 74)	74 (65, 79)	72 (65, 79)	72 (67, 77)
Gender, female, n (%)	176 (26)	70 (27.3)	44 (23.5)	6 (15.8)	6 (40)	2 (20)
Index year, n (%)						
2022	60 (8.9)	35 (13.7)	20 (10.7)	12 (31.6)	7 (46.7)	4 (40)
2023	616 (91.1)	221 (86.3)	167 (89.3)	26 (68.4)	8 (53.3)	6 (60)
Hemoglobin A1c, %						
Mean ± SD	7.3 ± 1.4	7.9 ± 1.8	7.7 ± 1.7	7.4 ± 1.2	7.7 ± 1.3	8.1 ± 1.4
Median (Q1, Q3)	7.1 (6.4, 7.9)	7.3 (6.8, 8.5)	7.3 (6.8, 8.3)	7.1 (6.5, 8)	7.4 (6.8, 8.3)	7.7 (7, 9.1)
Missing, n (%)	579 (85.7)	222 (86.7)	162 (86.6)	1 (2.6)	0 (0)	0 (0)
eGFR, mg/min/1.73 m^2^						
Mean ± SD	–	–	–	46.5 ± 20.8	37.3 ± 13.2	37.1 ± 17.0
Category, n (%)						
Stage 2 60–89	–	–	–	2 (5.3)	1 (6.7)	1 (10)
Stage 3 30–59	–	–	–	18 (47.4)	7 (46.7)	3 (30)
Stage 4 15–29	–	–	–	18 (47.4)	7 (46.7)	6 (60)
Finerenone dose initiation, n (%)						
10 mg	598 (88.5)	217 (84.8)	158 (84.5)	35 (92.1)	15 (100)	10 (100)
20 mg	78 (11.5)	39 (15.2)	29 (15.5)	3 (7.9)	0 (0)	0 (0)
Comorbidity, n (%)						
Hypertension	627 (92.8)	239 (93.4)	175 (93.6)	36 (94.7)	13 (86.7)	8 (80)
Hyperlipidemia	394 (58.3)	174 (68)	125 (66.8)	25 (65.8)	12 (80)	8 (80)
Congestive heart failure	425 (62.9)	148 (57.8)	111 (59.4)	24 (63.2)	9 (60)	6 (60)
Prior hospitalization for heart failure	171 (25.3)	58 (22.7)	43 (23.0)	7 (18.4)	1 (6.7)	1 (10)
Coronary heart disease	272 (40.2)	101 (39.5)	82 (43.9)	11 (29)	3 (20)	1 (10)
Peripheral vascular disease	87 (12.9)	46 (18)	31 (16.6)	6 (15.8)	4 (26.7)	1 (10)
Atrial fibrillation	118 (17.5)	22 (8.6)	16 (8.6)	7 (18.4)	1 (6.7)	0 (0)
Acute coronary syndrome	136 (20.1)	45 (17.6)	35 (18.7)	12 (31.6)	0 (0)	0 (0)
Myocardial infarction	70 (10.4)	19 (7.4)	16 (8.6)	7 (18.4)	0 (0)	0 (0)
Cerebrovascular disease	138 (20.4)	68(26.6)	40 (21.4)	10 (26.3)	3(20)	2 (20)
Neuropathy	138 (20.4)	65 (25.4)	48 (25.7)	4(10.5)	3(20)	2 (20)
Retinopathy	107 (15.8)	38 (14.8)	30 (16)	8 (21.1)	0 (0)	0 (0)
Charlson Comorbidity Index						
Mean ± SD	8.1 ± 3.1	8.1 ± 3.0	8.0 ± 3.0	9.7 ± 3.9	8.8 ± 2.8	8.4 ± 3.1
Median (Q1, Q3)	8 (6, 9)	8 (6, 10)	8 (6, 9)	9 (7, 11)	9 (7, 10)	8 (6, 10)
Diabetes Complication Severity Index						
Mean ± SD	5.2 ± 1.8	5.2 ± 1.7	5.1 ± 1.7	6.3 ± 2.1	6.1 ± 1.7	5.8 ± 1.3
Median (Q1, Q3)	6 (4, 6)	6 (4, 6)	5 (4, 6)	6 (5, 8)	6 (5, 7)	6 (5, 7)
Comedications, n (%)						
ACEi or ARB	553 (81.8)	213 (83.2)	158 (84.5)	31 (81.6)	11 (73.3)	6 (60)
ACEi	227 (33.6)	89 (34.8)	64 (34.2)	14 (36.8)	6 (40)	3 (30)
ARB	523 (77.4)	200 (78.1)	153 (81.8)	30 (79)	8 (53.3)	6 (60)
ARNI	184 (27.2)	52 (20.3)	46 (24.6)	9 (23.7)	1 (6.7)	1 (10)
Calcium-channel blockers	326 (48.2)	130 (50.8)	96 (51.3)	17 (44.7)	7 (46.7)	4 (40)
Beta-blockers	235 (34.8)	77 (30.1)	61 (32.6)	15 (39.5)	5 (33.3)	3 (30)
Loop diuretics	170 (25.2)	49 (19.1)	40 (21.4)	14 (36.8)	3 (20)	3 (30)
Thiazide diuretics	30 (4.4)	17 (6.6)	10 (5.4)	1 (2.6)	0 (0)	0 (0)
Steroidal MRA	117 (17.3)	29 (11.3)	22 (11.8)	3 (7.9)	0 (0)	0 (0)
Non-steroidal MRA other than finerenone	43 (6.4)	16 (6.3)	13 (7)	1 (2.6)	0 (0)	0(0)
Statins	410 (60.7)	169 (66)	128 (68.5)	27 (71.1)	11 (73.3)	7 (70)
Anticoagulants	114 (16.9)	30 (11.7)	22 (11.8)	8 (21.1)	1 (6.7)	0 (0)
Potassium binders	41 (6.1)	20 (7.8)	14 (7.5)	1 (2.6)	0 (0)	0 (0)
SGLT-2i and GLP-1 RA	192 (28.4)	192 (75)	183 (97.9) *	13 (34.2)	12 (80)	10 (100)
Metformin	218 (32.3)	112 (43.8)	88 (47.1)	13 (34.2)	6 (40)	5 (50)
Dipeptidyl peptidase 4 inhibitors	316 (46.8)	59 (23.1)	45 (24.1)	24 (63.2)	5 (33.3)	3 (30)
Sulfonylureas	88 (13)	40 (15.6)	31 (16.6)	9 (23.7)	3 (20)	3 (30)
Meglitinides	74 (11)	49 (19.1)	29 (15.5)	6 (15.8)	4 (26.7)	3 (30)
Alpha-glucosidase inhibitors	74 (11)	35 (13.7)	25 (13.4)	2 (5.3)	1 (6.7)	1 (10)
Thiazolidinediones	20 (3)	17 (6.6)	11 (5.9)	1 (2.6)	1 (6.7)	1 (10)
Insulins	196 (29)	147 (57.4)	101 (54)	10 (26.3)	7 (46.7)	5 (50)

* The number was not matched to the total number of the cohort because of different data collection period, i.e., n and % of concomitant use of SGLT-2i and GLP-1 RA as baseline covariates were assessed between day −365 and 0 (the index date), whereas the stratification of subgroups were based on the records between day −90 and 30. Abbreviations: ACEi, angiotensin-converting-enzyme inhibitor; ARB, angiotensin-receptor blocker; ARNI, angiotensin-receptor blocker/neprilysin inhibitor; CKD, chronic kidney disease; eGFR, estimated glomerular filtration rate; GLP-1 RA, glucagon-like peptide 1 receptor agonist; MRA, mineralocorticoid receptor antagonist; SD, standard deviation; and SGLT-2i, sodium–glucose cotransporter 2 inhibitor.

**Table 3:** Incidence of hyperkalemia during the follow-up after finerenone initiation. The results are presented in numbers of cases per person-years of follow-up. Abbreviations: MDV, Medical Data Vision database; RWD Co., Real World Data Co., Ltd. database.

	Number of Persons in Cohort	Number of Persons at Risk	Number of Events	Incidence Proportion (per 100 Persons)
MDV				
Hyperkalemia	967	832	18	2.16
Hospitalization associated with hyperkalemia	967	944	0	0
RWD Co.				
Hyperkalemia	62	37	1	2.70
Hospitalization associated with hyperkalemia	62	48	0	0

## Data Availability

The data included in this manuscript were used under contract with the providers (Medical Data Vision Co., Ltd. and Real World Data Co., Ltd.) and cannot be freely distributed by the authors. All necessary data required to interpret and conclude the findings of this study are included in the main text and Supplementary Materials.

## Supplementary Materials

The following supporting information can be downloaded at: https://www.mdpi.com/article/10.3390/jcm13175107/s1, Table S1. List of variables used to assess eligibility, comorbidities, and medications in the study. Table S2. Definition of clinical outcomes collected in the study [50,51,52,53]. Table S3. STROBE Checklist of the study [35]. Table S4. Baseline characteristics of the persons in the subset of individuals with congestive heart failure. Table S5. Incidence of cardiovascular composite clinical outcomes and kidney failure during the follow-up after finerenone initiation. Table S6. Incidence of clinical outcomes in the subset of individuals with congestive heart failure. Table S7. Sensitivity analysis of hyperkalemia occurred during the follow-up after finerenone initiation in overall study population and in the subset of individuals with congestive heart failure.
