# Supplementary material for: Early Clinical Experience of Finerenone in People with Chronic Kidney Disease and Type 2 Diabetes in Japan—A Multi-Cohort Study from the FOUNTAIN (FinerenOne mUltidatabase NeTwork for Evidence generAtIoN) Platform
Source: J Clin Med. 2024 Aug 28;13(17):5107. doi: 10.3390/jcm13175107 (PMC11396164; doi:10.3390/jcm13175107)
Supplement: Supplementary file 1 [file jcm-13-05107-s001.zip › jcm-3127716-supplementary.pdf]

SUPPLEMENTARY MATERIALS

Table S1. List of variables used to assess eligibility, comorbidities, and medications in the study……………2
Table S2. Definition of clinical outcomes collected in the study…………….…………………………………4
Table S3. STROBE Checklist of the study…………………………….…………………………………..6
Table S4. Baseline characteristics of the persons in the subset of individuals with congestive heart failure…8
Table S5. Incidence of cardiovascular composite clinical outcomes and kidney failure during the follow-up after finerenone initiation………………………………………………………………………….….…10
Table S6. Incidence of clinical outcomes in the subset of individuals with congestive heart failure….……..11
Table S7. Sensitivity analysis of hyperkalemia occurred during the follow-up after finerenone initiation in overall study population and in the subset of individuals with congestive heart failure…………………..12
References……………………..…………………………………………………………………………..11

Supplementary tables

Table S1. List of variables used to assess eligibility, comorbidities, and comedications in the study. Abbreviations: ACEi, angiotensin-converting-enzyme inhibitor; ARB, angiotensin-receptor blocker; ARNI, angiotensin-receptor blocker neprilysin inhibitor; CKD, chronic kidney disease; eGFR, estimated glomerular filtration rate; GLP-1 RA, glucagon-like peptide-1 receptor agonist; MRA, mineralocorticoid receptor antagonist; SGLT-2i, sodium-glucose cotransporter-2 inhibitor; UACR, urine albumin-to-creatinine ratio.

**Variables**	**Definition**
Variables to assess the eligibility	
Type 2 diabetes	Evidence of type 2 diabetes at any point before (and including) the index date.
CKD (stage)	Presence of following criteria at any point before (and including) the index date:<br>• A diagnosis code indicating CKD; **or**<br>• Two UACR test results ≥30 mg/g separated by at least 90 days and by not more than 540 days; **or**<br>• Two different eGFR test results ≥15 mL/min/1.73m^2 and <60 mL/min/1.73m^2 separated by at least 90 days and not more than 540 days.<br>• CKD stage was categorized based on the eGFR test results:<br>➢ Stage 2 eGFR ≥60 mL/min/1.73m^2 and <90 mL/min/1.73m^2<br>➢ Stage 3 eGFR ≥30 mL/min/1.73m^2 and <60 mL/min/1.73m^2<br>➢ Stage 4 eGFR ≥15 mL/min/1.73m^2 and <30 mL/min/1.73m^2
Kidney failure	Presence of following criteria at any point before (and including) the index date;<br>• Two different eGFR test results <15 mL/min/1.73m^2 separated by at least 90 days and by not more than 540 days; **or**<br>• Dependence of dialysis (at least 3 sessions over at least 90 days during the baseline period); or<br>• A diagnosis code indicating kidney failure or CKD stage 5; **or**<br>• Kidney transplant.
Comorbidities	
Hypertension	Any diagnosis record of hypertension between days -365 and 0.
Hyperlipidemia	Any diagnosis record of hyperlipidemia between days -365 and 0.
Congestive heart failure	Any diagnosis record of congestive heart failure between days -365 and 0.
Prior hospitalization for heart failure	Any inpatient diagnosis of congestive heart failure recorded between days -365 and 0.
Peripheral vascular disease	Any diagnosis record of peripheral vascular disease between days -365 and 0.
Coronary artery disease	Any diagnosis record of coronary artery disease between days -365 and 0.
Atrial fibrillation	Any diagnosis record of atrial fibrillation between days -365 and 0.
Acute coronary syndrome	Any diagnosis record of acute coronary syndrome between days -365 and 0.
Myocardial infarction	Any diagnosis record of myocardial infarction between days -365 and 0.
Cerebrovascular disease	Any diagnosis record of cerebrovascular disease between days -365 and 0.
Neuropathy	Any diagnosis record of diabetic neuropathy between days -365 and 0.
Retinopathy	Any diagnosis record of diabetic retinopathy between days -365 and 0.
Medications	
ACEi	Any prescription of ACEi recorded between days -180 and 0.

ARB	Any prescription of ARB recorded between days -180 and 0.
Calcium channel blocker	Any prescription of calcium channel blocker between days -180 and 0.
Beta-blocker	Any prescription of beta-blocker recorded between days -180 and 0.
Loop diuretics	Any prescription of loop diuretics recorded between days -180 and 0.
Thiazide diuretics	Any prescription of thiazide diuretics recorded between days -180 and 0.
Steroidal MRA	Any prescription steroidal MRA recorded between days -180 and 0.
Non-steroidal MRA	Any prescription of non-steroidal MRA other than finerenone recorded between days -180 and 0.
ARNI	Any prescription of ARNI recorded between days -180 and 0.
Statins	Any prescription of statin recorded between days -180 and 0.
Anticoagulants	Any prescription of anticoagulants recorded between days -180 and 0.
Potassium binder	Any prescription of potassium binder recorded between days -180 and 0.
SGLT-2i	Any prescription of SGLT-2i recorded between days -180 and 0.
GLP-1 RA	Any prescription of GLP-1 RA recorded between days -180 and 0.
Metformin	Any prescription of metformin recorded between days -180 and 0.
Dipeptidyl peptidase-4 inhibitors	Any prescription of dipeptidyl peptidase-4 inhibitor recorded between days -180 and 0.
Sulfonylureas	Any prescription of sulfonylureas recorded between days -180 and 0.
Meglitinides	Any prescription of meglitinides recorded between days -180 and 0.
Alpha glucosidase inhibitors	Any prescription of alpha glucosidase inhibitors recorded between days -180 and 0.
Thiazolidinediones	Any prescription of thiazolidinediones recorded between days -180 and 0.
Insulin	Any prescription of insulin recorded between days -180 and 0.

Table S2. Definitions of clinical outcomes collected in the study. Abbreviations: CKD, chronic kidney disease; eGFR, estimated glomerular filtration rate.

Clinical outcomes	Definition
Kidney failure	The fist occurrence of any of the following:<br>• ≥2 outpatient eGFR measurements of <15 mL/min/1.73m^2 separated by at least 90 days. eGFR determinations should be based on only outpatient measurements (inpatient measurements are more likely to be associated with transient changes in eGFR associated with acute events)<br>• Record of dependence on dialysis (at least 3 sessions over at least 90 days) [1]<br>• Diagnosis records of kidney failure or CKD stage 5<br>• Record of kidney transplant
Acute myocardial infarction	Inpatient hospital diagnosis of fatal or non-fatal acute myocardial infarction.
Congestive heart failure	Inpatient hospital or emergency department diagnosis of heart failure [2-4].
Cardiovascular composite outcome	Composite outcome of acute myocardial infarction and congestive heart failure.
Hyperkalemia	A set of at least one potassium level >5.5 mmol/L **or** at least one diagnostic code for hyperkalemia in the patient record.
Hospitalization associated with hyperkalemia	Record of hospitalization and the index date of a hyperkalemia event that occur in a time-period no more than 7 days apart. The second criterium (date of hospitalization date or date of hyperkalemia event) was considered the time of the "hospitalization associated with hyperkalemia" event.
Hyperkalemia (sensitivity analysis)	The earliest presence of<br>i. a set of at least two potassium values > 5.5 mmol/L or diagnostic code for hyperkalemia, specifically<br>• two serum potassium values >5.5 mmol/L on the inpatient record between 2 and 24 hours apart; **or**<br>• one serum potassium value >5.5 mmol/L for hyperkalemia on the outpatient record and one such value on the inpatient or outpatient record not longer than 7 days apart; **or**<br>ii. a set of one elevated serum potassium value in combination with pharmacotherapy or a diagnostic code for hyperkalemia:<br>• one serum potassium value >5.5 mmol/L or diagnostic code for hyperkalemia on either the inpatient or the outpatient record and the initiation of pharmacotherapy not longer 3 days apart, namely:<br>➢ Potassium binders<br>✧ Sodium polystyrene sulfonate<br>✧ Calcium polystyrene sulfonate<br>✧ Sodium zirconium cyclosilicate<br>✧ Patilomer; **or**<br>• an inpatient or outpatient potassium lab value >5.5 mmol/L or the occurrence of an inpatient or

outpatient diagnosis code for hyperkalemia not longer than 3 days apart.

iii. a set of at least two diagnostic codes for hyperkalemia namely:
- two diagnostic codes for hyperkalemia on the inpatient record between 2 and 24 hours apart, **or**
- one diagnostic code for hyperkalemia and one such value on the inpatient or outpatient record no longer than 7 days apart, **or**

iv. a set of one diagnostic code for hyperkalemia on either the inpatient or outpatient record and the initiation of specific pharmacotherapy for hyperkalemia no longer than 3 days apart, namely:
- ➢ Potassium binders
  - ✧ Sodium polystyrene sulfonate
  - ✧ Calcium polystyrene sulfonate
  - ✧ Sodium zirconium cyclosilicate
  - ✧ Patilomer

The date of the second event was defined as the date of hyperkalemia.

Table S3. STROBE (Strengthening the Reporting of Observational Studies in Epidemiology) Checklist of the study [5].

	Item No.	Recommendation	Page No.
**Title and abstract**	1	(*a*) Indicate the study design with a commonly used term in the title or abstract	1
		(*b*) Provide in the abstract an informative and balanced summary of what was done and what was found	2
**Introduction**			
Background/ rationale	2	Explain the scientific background and rationale for the investigation being reported	3
Objectives	3	State the specific objectives, including any prespecified hypotheses	3
**Methods**			
Study design	4	Present key elements of study design early in the paper	4
Setting	5	Describe the setting, locations, and relevant dates, including periods of recruitment, exposure, follow-up, and data collection	4
Participants	6	(*a*) *Cohort study*—Specify the eligibility criteria and the sources and methods of selection of participants. Describe methods of follow-up	4
		(*b*) For matched studies, provide matching criteria and the number of exposed and unexposed	N/A
Variables	7	Clearly define all outcomes, exposures, predictors, potential confounders, and effect modifiers. Give diagnostic criteria, if applicable	5
Data sources/ measurement	8	For each variable of interest, give sources of data and details of methods of assessment (measurement). Describe the comparability of assessment methods if there is more than one group	5
Bias	9	Describe any efforts to address potential sources of bias	9
Study size	10	Explain how the study size was arrived at	6
Quantitative variables	11	Explain how quantitative variables were handled in the analyses. If applicable, describe which groupings were chosen and why	5
Statistical methods	12	(*a*) Describe all statistical methods, including those used to control for confounding	5
		(*b*) Describe any methods used to examine subgroups and interactions	4
		(*c*) Explain how missing data were addressed	5
		(*d*) If applicable, explain how the loss to follow-up was addressed	N/A
		(e) Describe any sensitivity analyses	5

Continued on the next page

**Results**			
Participants	13	(a) Report numbers of individuals at each stage of study—e.g., numbers potentially eligible, examined for eligibility, confirmed eligible, included in the study, completing follow-up, and analyzed	6
		(b) Give reasons for non-participation at each stage	6
		(c) Consider the use of a flow diagram	6
Descriptive data	14	(a) Give characteristics of study participants (e.g., demographic, clinical, social) and information on exposures and potential confounders	6
		(b) Indicate the number of participants with missing data for each variable of interest	13
		(c) Summarize follow-up time (e.g., average and total amount)	7
Outcome data	15	Report numbers of outcome events or summary measures over time	17
Main results	16	(*a*) Give unadjusted estimates and, if applicable, confounder-adjusted estimates and their precision (e.g., 95% confidence interval). Make clear which confounders were adjusted for and why they were included	14
		(*b*) Report category boundaries when continuous variables were categorized	14
		(*c*) If relevant, consider translating estimates of relative risk into absolute risk for a meaningful time period	N/A
Other analyses	17	Report other analyses performed—e.g., analyses of subgroups and interactions and sensitivity analyses	7, 17
**Discussion**			
Key results	18	Summarize key results with reference to study objectives	8
Limitations	19	Discuss the limitations of the study, taking into account sources of potential bias or imprecision. Discuss both the direction and magnitude of any potential bias	9
Interpretation	20	Give a cautious overall interpretation of results considering objectives, limitations, the multiplicity of analyses, results from similar studies, and other relevant evidence	7-9
Generalizability	21	Discuss the generalizability (external validity) of the study results	9
**Other information**			
Funding	22	Give the source of funding and the role of the funders for the present study and, if applicable, for the original study on which the present article is based	10

Table S4. Baseline characteristics of the persons in the subset of individuals with congestive heart failure. Abbreviations: ACEi, angiotensin-converting enzyme inihibitor; ARB, angiotensin-receptor blocker; ARNI, angiotensin-receptor blocker neprilysin inhibitor; CKD, chronic kidney disease; eGFR, estimated glomerular filtration rate; GLP-1 RA, glucagon-like peptide-1 receptor agonist; IQR, interquartile range; MRA, mineralocorticoid receptor antagonist; SD, standard deviation; SGLT-2i, sodium-glucose cotransporter-2 inhibitor; and UACR, urine albumin-to-creatinine ratio.

	**MDV (N = 508)**	**RWD (N = 33)**
Age (years)		
Mean ± SD	71.6 ± 12.2	72.9 ± 11.9
Median (IQR)	74 (65–80)	76 (65–82)
Gender, female, n (%)	124 (24.4)	10 (30.3)
Index year, n (%)		
2022	50 (9.8)	10 (30.3)
2023	458 (90.2)	23 (69.7)
Hemoglobin A1c, %		
Mean ± SD	7.1 ± 14	7.5 ± 1.2
Median (IQR)	6.8 (6.2–7.6)	7.3 (6.6–8.3)
Missing, n (%)	447 (88.0)	0 (0)
Systolic blood pressure, mmHg		
Mean ± SD	–	134.6 ± 21.7
Diastolic blood pressure, mmHg		
Mean ± SD	–	76.3 ± 9.2
UACR, mg/g		
Mean ± SD	–	181.3 ± 128.6
Median (IQR)	–	150 (30–300)
Category, n (%)		
≥0 and <30	–	4 (12.1)
≥30 and <300	–	9 (27.3)
≥300	–	5 (15.2)
Missing	–	15 (45.5)
eGFR, mg/min/1.73m^2		
Mean ± SD	–	43.1 ± 17.1
Category, n (%)		
Stage 2 60–89	–	0 (0)
Stage 3 30–59	–	16 (48.5)
Stage 4 15–29	–	15 (51.5)
Finerenone dose at initiation, n (%)		
10 mg	454 (89.4)	30 (90.9)
20 mg	54 (10.6)	3 (9.1)
Comorbidity, n (%)		
Hypertension	482 (94.9)	32 (97.0)
Hyperlipidemia	322 (63.4)	22 (66.7)
Congestive heart failure	508 (100)	33 (100)
Prior hospitalization for heart failure	206 (40.6)	10 (30.3)
Coronary heart disease	274 (53.9)	17 (51.5)
Peripheral vascular disease	83 (16.3)	11 (33.3)
Atrial fibrillation	139 (27.4)	10 (30.3)
Acute coronary syndrome	147 (28.9)	11 (33.3)
Myocardial infarction	78 (15.4)	8 (24.2)
Cerebrovascular disease	134 (26.4)	8 (24.2)
Neuropathy	94 (18.5)	5 (15.2)
Retinopathy	64 (12.6)	2 (6.1)
Charlson Comorbidity Index		

Mean ± SD	8.8 ± 3.1	10.5 ± 3.8
Median (IQR)	8 (7–10)	10 (8–12)
Diabetes Complication Severity Index		
Mean ± SD	5.8 ± 1.7	6.8 ± 1.9
Median (IQR)	6 (4–6)	6 (6–8)
Medications, n (%)		
ACEi or ARB	418 (82.3)	25 (75.8)
ACEi	168 (33.1)	15 (45.5)
ARB	396 (78.0)	22 (66.7)
ARNI	168 (33.1)	7 (21.2)
Calcium channel blockers	257 (50.6)	16 (48.5)
Beta-blockers	249 (49.0)	18 (54.6)
Loop diuretics	186 (36.6)	12 (36.4)
Thiazide diuretics	18 (3.5)	1 (3.0)
Steroidal MRA	132 (26.0)	5 (15.2)
Non-steroidal MRA other than finerenone	35 (6.9)	1 (3.0)
Statins	310 (61.0)	20 (60.6)
Anticoagulants	133 (26.2)	11 (33.3)
Potassium binders	37 (7.3)	1 (3.0)
SGLT-2i	393 (77.4)	24 (72.7)
GLP-1 RA	135 (26.6)	10 (30.3)
SGLT-2i or GLP-1 RA	420 (82.7)	26 (78.8)
SGLT-2i and GLP-1 RA	108 (21.3)	8 (24.2)
Metformin	131 (25.8)	12 (36.4)
Dipeptidyl peptidase 4 inhibitors	228 (44.9)	22 (66.7)
Meglitinides	61 (12.0)	6 (18.2)
Sulfonylureas	59 (11.6)	6 (18.2)
Alpha glucosidase inhibitors	48 (9.5)	3 (9.1)
Thiazolidinediones	11 (2.2)	1 (3.0)
Insulins	156 (30.7)	11 (33.3)

Table S5. Incidence of cardiovascular composite clinical outcomes and kidney failure during the follow-up after finerenone initiation. The results are presented in numbers of cases per person-years of follow up. Abbreviations: CHF, congestive heart failure; MDV, Medical Data Vision; MI, myocardial infarction; RWD, real-world data.

	**Number of persons in cohort**	**Number of persons at risk**	**Number of events**	**Incidence proportion (per 100 cases)**
MDV				
Cardiovascular composite outcome	967	875	6	0.69
MI	967	733	12	1.64
CHF	967	860	3	0.35
Kidney failure	967	743	12	1.62
RWD				
Cardiovascular composite outcome	62	35	0	0
MI	62	38	0	0
CHF	62	36	0	0
Kidney failure	62	39	0	0

Table S6. Incidence of clinical outcomes during the follow-up after finerenone initiation in the subset of individuals with congestive heart failure. The results are presented in numbers of cases per person-years of follow up. Abbreviations: CHF, congestive heart failure; MDV, Medical Data Vision; MI, myocardial infarction; RWD Co., real-world data.

	Number of persons in cohort	Number of persons at risk	Number of events	Incidence proportion (per 100 persons)
MDV				
Cardiovascular composite outcome	508	289	8	2.77
MI	508	420	3	0.71
CHF	508	298	8	2.68
Kidney failure	508	464	3	0.65
Hyperkalemia	508	422	10	2.37
Hospitalization associated with hyperkalemia	508	494	0	0
RWD Co.				
Cardiovascular composite outcome	33	18	0	0
MI	33	20	0	0
CHF	33	18	0	0
Kidney failure	33	19	0	0
Hyperkalemia	33	20	1	5.00
Hospitalization associated with hyperkalemia	33	27	0	0

Table S7. Sensitivity analysis of hyperkalemia occurred during the follow-up after finerenone initiation in overall study population and in the subset of individuals with congestive heart failure. The results are presented in numbers of cases per person-years of follow up. Abbreviations: CHF, congestive heart failure; MDV, Medical Data Vision; RWD Co., real-world data.

	Number of persons in cohort	Number of persons at risk	Number of events	Incidence proportion (per 100 persons)
MDV				
Overall	967	832	8	2.16
CHF subset	508	422	0	2.37
RWD Co.				
Overall	62	38	1	2.63
CHF subset	33	21	1	4.76

References

1. Ruilope LM, Agarwal R, Anker SD, Bakris GL, Filippatos G, Nowack C, et al. Design and baseline characteristics of the finerenone in reducing cardiovascular mortality and morbidity in diabetic kidney disease trial. *Am J Nephrol.* 2019; **50**(5): 345-356.

2. Sundbøll J, Adelborg K, Munch T, Frøslev T, Sørensen HT, Bøtker HE, et al. Positive predictive value of cardiovascular diagnoses in the Danish National Patient Registry: a validation study. *BMJ Open.* 2016; **6**(11): e012832.

3. Maru S, Koch GG, Stender M, Clark D, Gibowski L, Petri H, et al. Antidiabetic drugs and heart failure risk in patients with type 2 diabetes in the U.K. primary care setting. *Diabetes Care.* 2005; **28**(1): 20-26.

4. Saczynski JS, Andrade SE, Harrold LR, Tjia J, Cutrona SL, Dodd KS, et al. A systematic review of validated methods for identifying heart failure using administrative data. *Pharmacoepidemiol Drug Saf.* 2012; **21**(S1): 129-140.

5. von Elm E, Altman DG, Egger M, Pocock SJ, Gøtzsche PC, Vandenbroucke JP, et al. The Strengthening the Reporting of Observational Studies in Epidemiology (STROBE) Statement: guidelines for reporting observational studies. *Ann Int Med.* 2008; **148**(2): 573-577
